# Investigation of Rheological and Flow Properties of Buckwheat Dough with and Without Xanthan and Guar Gums for Optimized 3D Food Printing Across Temperature Variations

**DOI:** 10.3390/foods13244054

**Published:** 2024-12-16

**Authors:** Sholpan Baimaganbetova, Sagyn Omirbekov, Yanwei Wang, Mei-Yen Chan, Didier Talamona

**Affiliations:** 1 Department of Mechanical and Aerospace Engineering, School of Engineering and Digital Sciences, Nazarbayev University, Astana 010000, Kazakhstan; 2 Center for Energy and Advanced Materials Science, National Laboratory Astana, Nazarbayev University, Astana 010000, Kazakhstanyanwei.wang@nu.edu.kz (Y.W.); 3 Department of Chemical and Materials Engineering, School of Engineering and Digital Sciences, Nazarbayev University, Astana 010000, Kazakhstan; 4 Department of Biomedical Sciences, School of Medicine, Nazarbayev University, Astana 010000, Kazakhstan; 5 Institute of Smart Systems and Artificial Intelligence (ISSAI), Nazarbayev University, Astana 010000, Kazakhstan

**Keywords:** sustainable plant protein sources, buckwheat, customizable food design, rheological properties, bioprinting for food, personalized food fabrication

## Abstract

Buckwheat (*Fagopyrum esculentum*) is a gluten-free crop valued for its protein, fiber, and essential minerals. This study investigates the rheological properties of buckwheat (BW) dough, both with and without the addition of gums (no gum, guar (GG), xanthan (XG)), at varying barrel temperatures (25, 55, and 85 °C) of the rheometer and at different water content levels (45, 50, and 55% *w*/*w*) to optimize dough formulations for 3D food printing. Using high shear stress capillary tests, the consistency coefficient (K) and flow behavior index (n) were measured. The results indicated that GG significantly increases the apparent viscosity of buckwheat dough across shear rates ranging from 200 to 2000 s^−1^, under all temperature and water content conditions. XG also enhanced viscosity but to a lesser extent at moderate temperatures (55 °C, 85 °C). All BW dough formulations exhibited a non-Newtonian shear-thinning behavior, crucial for 3D printing applications. In addition, computational fluid dynamics (CFD) simulations were conducted to analyze the extrusion process of BW dough formulations (50% W, 50% W + XG, and 50% W + GG), focusing on shear rate, viscosity, and pressure distribution. The simulations demonstrated that shear rates increased as the dough moved through the nozzle, while viscosity decreased, facilitating extrusion. However, gum-added formulations required higher pressures for extrusion, indicating an increased difficulty in dough flow. The study highlights the complex interactions between temperature, water content, and additive type on the rheological properties of buckwheat dough, while also incorporating CFD simulations to analyze the extrusion process. These insights provide a foundation for developing nutrient-dense, gluten-free 3D-printed foods tailored to specific dietary needs.

## 1. Introduction

Buckwheat (*Fagopyrum esculentum*) is a fast-maturing, gluten-free crop known for its nutritious seeds and ability to germinate in infertile soils and under a variety of environmental conditions [1]. Buckwheat (BW) is packed with vital nutrients like protein, dietary fiber, and key minerals such as magnesium and phosphorus, making it suitable for individuals with celiac disease or gluten sensitivity [2,3]. It flourishes in nutrient-poor soils and harsh climates, making it an excellent crop for the diverse and challenging conditions found in Central Asia [4]. In Central Asia, BW is a staple food, commonly used in local dishes such as porridge and pancakes, with its flour being essential in regional cuisines [5]. The crop’s sustainability and nutritional benefits are pivotal for food security in the area.

Consuming meat increases the risks of heart disease and cancer, and it negatively impacts the environment due to high greenhouse gas emissions and its resource-heavy production. Plant-based meats, as viable alternatives, offer similar taste and nutrition while providing reduced health risks and a smaller environmental footprint [6,7]. BW’s high protein content and beneficial amino acid profile make it an excellent candidate for plant-based meat formulations, enhancing both the nutritional value and texture of these products [8,9].

3D printing with BW has shown limited but promising applications, especially in the food sector. Researchers have explored using BW flour to create customized, nutrient-dense foods tailored to specific dietary needs. For instance, buckwheat flour has been effectively used in 3D food printing to develop personalized plant-based substitutes for traditional meat, which are both nutrient-rich and customized to individual dietary requirements [10]. Additionally, BW-based pastes have been used to print complex, edible structures, opening new avenues for the food industry to produce novel and functional foods that meet specific consumer needs [11]. More research is needed to optimize the formulations of BW pastes and enhance their printability and structural integrity. Understanding the rheological properties of BW-based materials is crucial in improving their performance in 3D printing applications, with the potential to lead to more robust and versatile 3D-printed food products [12]. There is a significant opportunity to develop 3D-printed foods combining BW with other functional ingredients, creating innovative, health-promoting products that offer both nutritional and functional benefits [13].

Understanding the rheological properties of BW dough is crucial for successful 3D food printing. Rheological properties determine dough behavior during printing, affecting extrusion and layer formation [14]. Consistent rheological behavior ensures uniform texture and structural integrity in printed products. As the dough from BW flour is crumblier than dough from other cereals, it is desirable to add gum to improve water retention [15]. Adding gums such as guar gum (GG) and xanthan gum (XG) significantly impacts rheological properties. GG and XG are preferred over other types of gums for BW dough because of their affordability, natural source availability, and ability to enhance texture, increase water retention, stabilize emulsions, and maintain product stability under various conditions. GG enhances viscosity and water-binding capacity, improving stability and reducing syneresis [16]. XG provides shear-thinning properties, allowing the dough to flow easily under pressure while maintaining its shape after extrusion [17].

To our knowledge, previous studies [18,19,20] have investigated the effects of BW flour concentration and different types of gums or proteins on the rheological and physical properties of gluten-free cakes. However, they did not consider the impact of temperature on these properties. To fill this gap, our study examines the complex interactions between temperature, water content, and additive type on the rheological properties of BW flour dough, utilizing high shear stress capillary tests.

Computational fluid dynamics (CFD) is crucial for understanding and optimizing extrusion processes in food manufacturing, especially in 3D food printing. Oyinloye and Yoon [21] highlighted the application of CFD simulation for effective food 3D printing design. Numerous studies have effectively employed CFD to analyze the flow characteristics of various food materials during extrusion. For instance, Singh and Muthukumarappan [22] simulated the flow of soy white flakes-based dough in single-screw extruders, modeling various screw speeds (40–200 RPM), barrel temperatures (100–140 °C), and soy contents (30–50% dry basis). Their simulations demonstrated that higher screw speeds increased shear rates and influenced viscosity, offering essential insights for optimizing extrusion processes and enhancing product quality. In another study, Oyinloye and Yoon [23] utilized CFD to optimize the printability of rice paste in 3D printing. They found that a rice-to-water ratio of 100:80 and controlled ambient temperature (47 ± 5 °C) improved structural stability. Their simulations indicated that smaller nozzle diameters increased die swell and pressure, affecting flow properties and deformation. Additionally, Guo et al. [24] conducted a comparative study of screw and syringe-based 3D printers through computational simulations. Their analysis revealed that screw-based printers exhibited complex fluid dynamics, including backflows between the walls and screw flights, complicating material flow. Conversely, syringe-based printers demonstrated simpler fluid characteristics, enabling easier adjustments during operation. This study also highlighted the limitations of screw-based systems in extruding high-viscosity inks, suggesting that these systems may not be suitable for certain formulations. Overall, integrating CFD into food extrusion research helps optimize processing conditions and improve product quality. This study aims to apply these principles to BW dough formulations to better understand their rheological properties and extrusion dynamics, contributing to the development of nutrient-dense 3D-printed foods.

In this study, the rheological and flow characteristics of BW dough were investigated to optimize its formulation for 3D food printing. Dough samples were prepared with varying water content (45, 50, and 55% *w*/*w*) and tested with and without the addition of gums (XG and GG). Shear stress and apparent viscosity were measured using a capillary rheometer at temperatures of 25, 55, and 85 °C. The rheological data, including the consistency coefficient (K) and the flow behavior index (n), were then used in CFD simulations, which were conducted at 25 °C. The geometry of the CFD model was based on a syringe-type 3D printer nozzle, and the simulations modeled the flow of dough through the nozzle, examining parameters such as pressure, apparent viscosity, and shear rate to predict printing performance and evaluate the effects of formulation and operating conditions. This combined experimental and computational approach enabled a thorough analysis of the factors affecting dough printability and its mechanical properties.

## 2. Materials and Methods

### 2.1. Materials

The dough ingredients included brown buckwheat (BW) flour (Garnec brand, Vladimir, Russia), containing 12.5% protein, 2.5% fat, and 70% carbohydrates; xanthan gum (XG) derived from *Xanthomonas campestris* with molecular weight ranging approximately from 1 × 10^6^ to 50 × 10^6^ Da (Sigma-Aldrich, St. Louis, MO, USA); guar gum (GG) derived from *Cyamopsis tetragonoloba* with molecular weight ranging approximately from 220,000 to 300,000 Da (Sigma-Aldrich, St. Louis, MO, USA); and distilled water.

### 2.2. Dough Preparation

The required amounts of BW flour, distilled water, and gums were measured according to Table 1. The BW flour and distilled water were thoroughly mixed and kneaded for samples without gum until a homogeneous consistency was achieved. For samples containing gum, 1% *w*/*w* of gum (XG or GG) was added to the measured amount of distilled water. The gum concentration was selected based on its widely recognized effective range in food formulations, as it helps enhance viscosity and water retention without adversely affecting the BW dough’s consistency or taste [20]. This gum–water mixture was then mixed using a mechanical stirrer (ISOLAB Laborgeräte GmbH, Eschau, Germany) at 1500 RPM for 5 min. Subsequently, the BW flour was gradually introduced into the gum–water mixture while stirring to form a dough. The dough was then kneaded until a homogeneous consistency was achieved. To prevent moisture loss, the dough was prepared immediately before each experiment.

A full factorial design (F2L3) was employed, encompassing the following two factors: water content (45, 50, and 55% *w*/*w*) [25] and gum inclusion (no gum, XG, GG), each at three levels (Table 1). In Table 1, the sample designations indicate dough formulations using the following abbreviations: “W” for water, followed by gum abbreviations indicating absence (-) or presence (“XG” or “GG”) in the dough. This resulted in 9 unique combinations, ensuring a comprehensive analysis of factor interactions. We added 1% *w*/*w* of gum per total mass of dough for experiments that contain gum. Each dough sample was maintained at a total mass of 50 g.

### 2.3. Rheological Measurements

In this study, an advanced twin-bore Rosand RH10 capillary rheometer (Malvern Instruments, Malvern, Worcester, UK) with Flowmaster^®^ version 8.3.10 control software [26] was utilized to evaluate the rheological properties of the dough material by extruding it through a capillary die under controlled conditions [27]. A cylindrical capillary die of known diameter, D = 1 mm, and length, L = 20 mm, was mounted at the bottom of the barrel. The dough material was loaded into one barrel before each experiment. A piston with a diameter of 15 mm was used to drive the dough through the die at various velocities corresponding to each shear rate. Precompression pressures of 0.5–1.0 MPa were applied during dough loading to the barrel to ensure uniform material distribution and eliminate air pockets, thereby minimizing particle migration and wall slip. The Bagley and Rabinowitsch corrections [28,29,30] further refined the pressure drop and shear rate calculations, ensuring the accurate characterization of the non-Newtonian flow behavior. The barrel preheated until the desired temperatures were reached, and the Flowmaster^®^ software recorded raw data.

**Figure 1:**
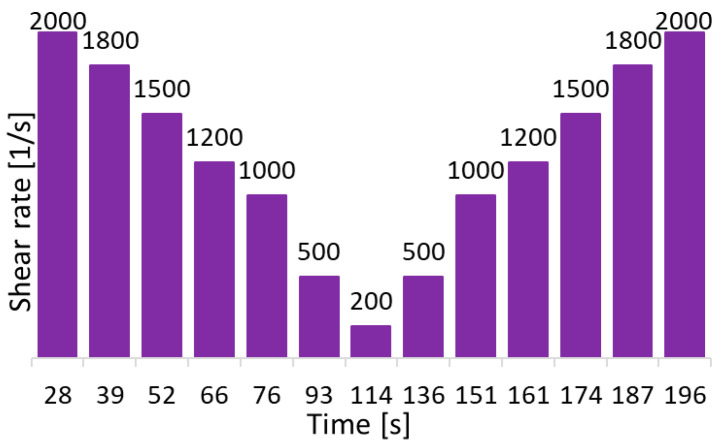
Shear rate steps in the rheometer.

The tests were conducted over a range of apparent shear rates through the die. As shown in Figure 1, the shear rate steps in the rheometer were set at 2000, 1800, 1500, 1200, 1000, 500, and 200 s^−1^. Then, they were reversed symmetrically to 500, 1000, 1200, 1500, 1800, and 2000 s^−1^. These shear rates fall within the range suitable for high-shear processes, such as those encountered in a 3D food printer [31]. This corresponds to the extrusion region within the typical ranges of shear rates for common industrial processes, as shown by Carnicer et al. [32]. This indicates that the selected shear rates are appropriate for studying the dough’s behavior under conditions similar to those in a 3D printing nozzle. Additionally, tests were conducted at three different barrel temperatures (25, 55, and 85 °C) for each of the 9 dough formulations, resulting in a total of 27 experiments. All reported measurements were triplicated for each sample to check their reproducibility. The deviations from the three repetitive tests are shown as error bars.

A capillary rheometer is a pressure-driven instrument used to simulate the flow of materials through a narrow die, typically resembling the geometry of an extruder die. It is designed to measure the rheological properties of materials, such as dough, under the shear rates and temperatures encountered during processing. [33]. The data for shear stress and shear rate were fitted to a power law equation, similar to the approach used in prior research on food materials under extrusion conditions, expressed by Equation (1) as follows [34]:

(1) $$\eta =K\cdot {\dot{\gamma }}^{\left(n-1\right)}$$

where η, $\dot{\gamma }$, K, and n represent the shear viscosity (Pa·s), shear rate ($s^{-1}$), consistency index ($Pa\cdot s^{n}$), and flow behavior index, respectively. The parameter n is indicative of the slope of the viscosity versus the shear rate curve. When n=1, the fluid exhibits Newtonian behavior, where viscosity remains constant regardless of the shear rate. As n deviates more from 1, the material shows greater pseudoplasticity [35]. Lower values of n suggest a significant reduction in viscosity with an increase in shear rate, indicating higher pseudoplasticity and greater structural complexity, which is advantageous for applications such as 3D printing. Since 3D printing technology involves the flow of material, it is crucial to study the rheological properties of the products intended for printing [36]. For food materials to be ideal for 3D printing, their viscosity must be sufficiently low to allow for smooth extrusion through a small nozzle, yet high enough to maintain cohesiveness and prevent deformation when layered on previously deposited material [37]. As a result, this property of food materials exhibits shear-thinning behavior.

### 2.4. CFD Simulation of the Extrusion Process

**Figure 2:**
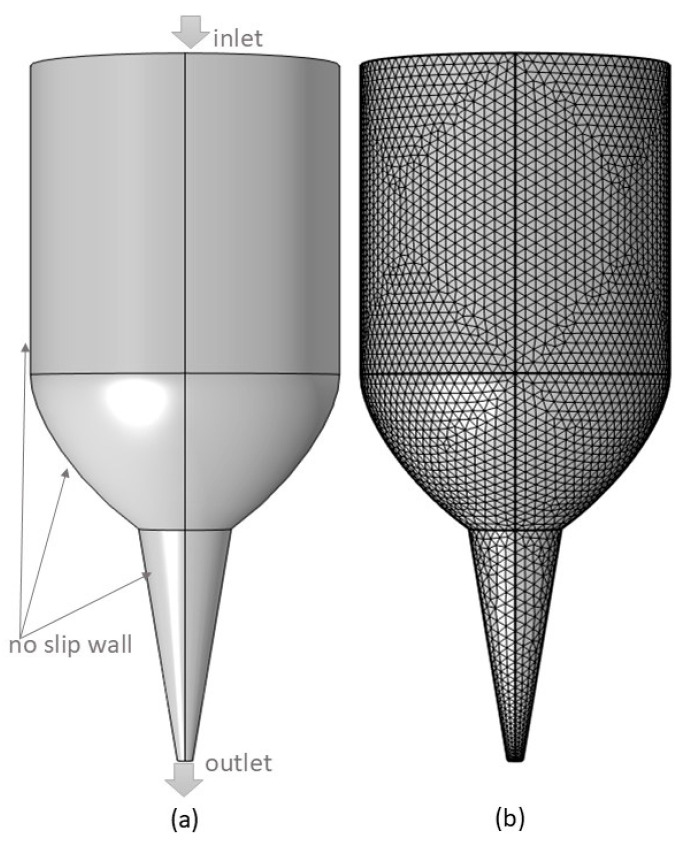
(**a**) Computer-Aided Design (CAD) model of the syringe tube used in syringe-based 3D printing, highlighting the essential boundary conditions for the flow domain; (**b**) meshing of the syringe geometry.

A simulation of the pseudoplastic flow related to syringe-based additive manufacturing (AM) was conducted using finite element method (FEM) software, COMSOL Multiphysics 6.2 (COMSOL Multiphysics, Burlington, VT, USA). In this study, the simulation was carried out only for the 50% W, 50% W + GG, and 50% W + XG dough formulations at a temperature of 25 °C. The size and shape of the syringe were selected based on the specifications of the Foodini [38] 3D printer. To optimize computational efficiency, the simulation domain was confined to the nozzle and a portion of the syringe [20], with a total length of 45 mm from the nozzle tip. The geometry used for the pseudoplastic flow simulation is depicted in Figure 2a, with a nozzle tip diameter of 1 mm. A tetrahedral mesh was applied to discretize the fluid domain within the geometry (Figure 2b), resulting in a total of 149,475 elements in the fluid region. The simulation was configured under steady-state and isothermal conditions, with the material property data appropriately defined. Based on the mesh setup, the model required a total of 209 s to compute the laminar flow behavior within the syringe tube.

The dough formulations were treated as an incompressible [23] single-phase fluid with laminar flow characteristics. The non-Newtonian behavior was modeled using a power law equation (Equation (1)), with the relevant parameters outlined in Table 2. To solve the governing conservation equations, the following continuity and momentum conservation equations were employed:

(2) ∇·v=0

(3) $$\rho \left(\frac{\partial v}{\partial t}+v\cdot \nabla v\right)=-\nabla p+\nabla \cdot \sigma$$

where v represents the velocity vector, ρ represents the density of the printing material, and p represents pressure. In Equation (3), gravitational forces are neglected because the viscous forces are significantly higher than the inertial forces in the system [39]. Moreover, σ represents the stress tensor, which is given by the following constitutive equation [35,40]:

(4) σ=2ηD

where η represents the shear-dependent non-Newtonian viscosity, and D denotes the rate of the deformation tensor of the fluid and follows the form given below:

(5) $$D=\frac{\nabla v+{\left(\nabla v\right)}^{T}}{2}$$

where ∇v is the velocity gradient tensor, and ${\left(\nabla v\right)}^{T}$ denotes the transpose of the velocity gradient tensor. Additionally, the following assumptions, initial conditions, and boundary conditions concerning the flow characteristics of the various dough formulations were applied: (i) the dough is fully contained within the syringe; (ii) the inlet flow rate is set to $30\;{\mathrm{mm}}^{3}\text{/}s$ to align with the parameters used in the printing experiments; (iii) there is no slip at the syringe wall ($v_{wall}=0$) during the extrusion process [37]; and (iv) the dough is initially stationary, with an initial velocity of 0 mm/s.

## 3. Results and Discussion

### 3.1. Apparent Viscosity and Effect of Water Content

**Figure 3:**
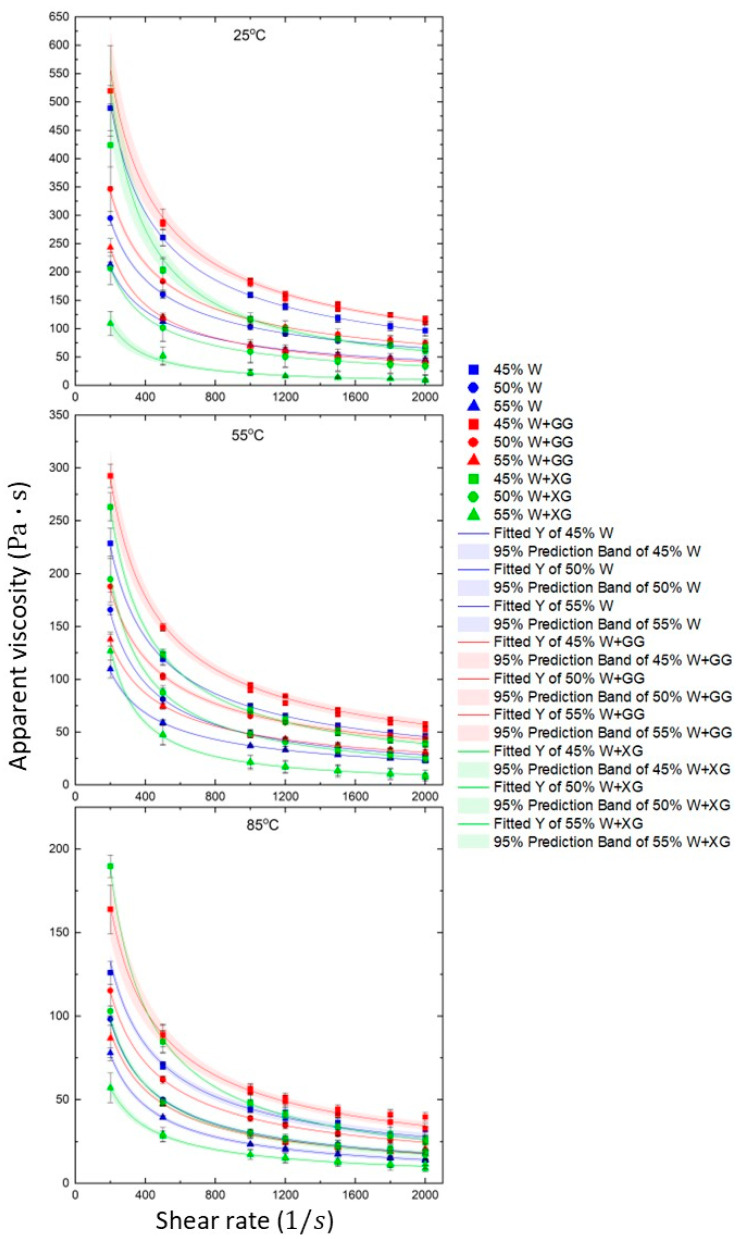
Experimental flow points for BW dough formulations at different temperatures with predicted curves from the power law model.

Since 3D printing technology involves the flow of material, it is crucial to study the flow behavior of the BW dough intended for printing [36]. Figure 3 illustrates the relationship between apparent viscosity and shear rate for different BW dough formulations and barrel temperatures. All curves show a sharp decrease in apparent viscosity at lower shear rates, which levels off at higher shear rates. This behavior indicates non-Newtonian shear thinning, where the apparent viscosity decreases as the shear rate increases. This pseudoplastic flow behavior is consistent with findings by other researchers in the context of food printing [41,42]. Each data point includes error bars representing the standard deviation, indicating the variability between the three replicates. Consistent error bars across the shear rates suggest that the measurements are reliable and the observed trends are significant.

Each curve in each graph represents different water concentrations (45, 50, and 55% *w*/*w*). The 45% *w*/*w* (square) water contents have the highest viscosity, followed by 50% *w*/*w* (circle), and with the 55% *w*/*w* (triangle) water content having the lowest viscosity. In all cases, increasing the concentration of water decreases the apparent viscosity. Several groups have reported comparable findings [43,44]. This suggests that water acts as a diluent, reducing the overall viscosity of the dough.

The shear rate was applied in a stepwise manner, starting from 2000 s^−1^ and decreasing to 200 s^−1^. After reaching 200 s^−1^, the shear rate increased symmetrically back to 2000 s^−1^. This method is often used to assess the hysteresis effect, where the material’s response to increasing and decreasing shear rates can be compared [45]. In this work, the hysteresis loop between the forward and backward shear rate points was observed to be insignificant in all samples and overlapped around the fitted curves. In the study by Lokumcu Altay and Ak [46], the reported hysteresis in the flow curves of tahini (sesame paste) was between 20 and 70 °C; however, they repeated the shear rate cycle twice to check whether the loop was preserved. In our measurements for BW dough in different formulations, looping was not strongly observed from the first cycle at 25, 55, and 85 °C. The absence of a hysteresis loop in the apparent viscosity data points across the shear rates indicates consistent measurements, suggesting that the material exhibits a reproducible shear-thinning behavior irrespective of the direction of shear rate application. This characteristic is crucial during the process of 3D food printing. In 3D food printing, the material needs to transition smoothly from a high-viscosity state (at low shear rates) to a low-viscosity state (at high shear rates) as it is extruded through the nozzle. The predictable decrease in viscosity with an increase in the shear rate ensures that the material can be extruded easily without clogging the nozzle, while maintaining sufficient viscosity to hold its shape once deposited.

### 3.2. Effect of Temperature on Buckwheat Dough Rheology

**Figure 4:**
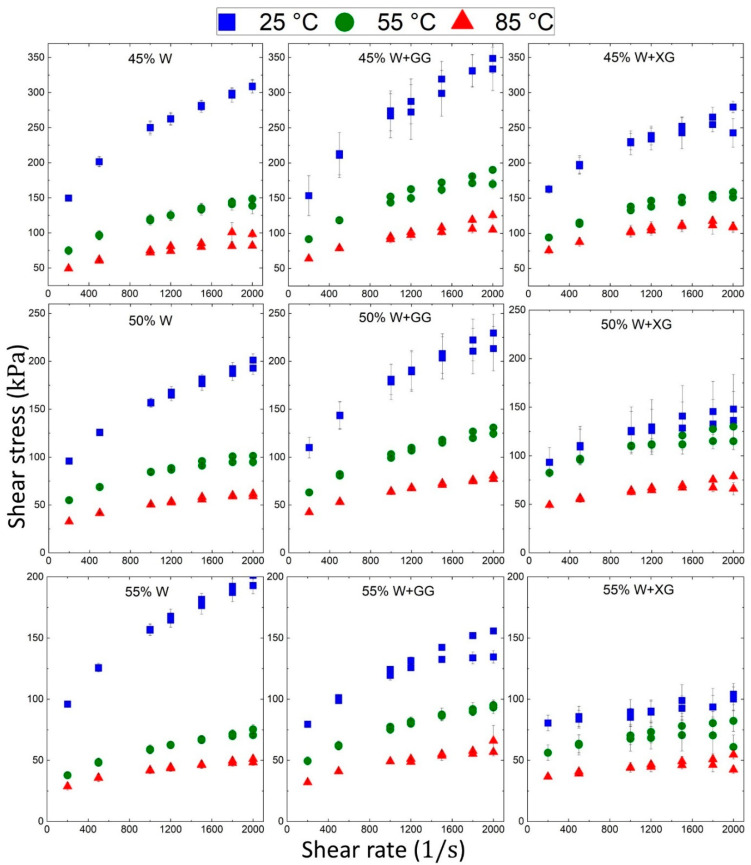
Effect of temperature and additives on shear stress versus shear rate for different buckwheat dough formulations.

Figure 4 illustrates the shear stress versus shear rate for different dough formulations, with data points color-coded to represent measurements at 25, 55, and 85 °C (red, green, and blue, respectively). The temperature has a noticeable impact on the rheological properties of the samples, with the extent of this impact depending on the composition of the dough [47]. The primary factor in Figure 4 is the effect of temperature on the shear stress of the samples. Higher temperatures consistently reduce the shear stress across all dough formulations, indicating that the material becomes less resistant to flow (i.e., less viscous) as temperature increases [48].

The sample with 45% W shows a clear decrease in shear stress with increasing temperature. The shear stress increases nearly linearly with the shear rate. The sample with 45% W + GG exhibits a higher shear stress than both 45% W and 45% W + XG under the same conditions. The sample with 45% W + XG shows similar behavior to 45% W + GG, but with lower shear stress values than 45% W, suggesting that XG does not increase the viscosity compared to 45% W. This is due to the conditions of the experiment, especially the condition of the stirrer rotation speed [49]. Samples with 50 and 55% W, with and without additives, show similar trends, with higher shear stresses compared to 45% W samples. The differences between additives (GG and XG) and the base substance follow the same pattern.

After loading the dough formulations into the barrel and precompressing them, the dough was left to rest for 5 min before testing. Inconsistent temperature distribution within the dough during this period may have prevented the full activation of temperature-sensitive processes, such as protein degradation and starch gelatinization, which typically increase viscosity. This could explain the lack of significant viscosity increase at 55 °C and 85 °C. The heating (55 °C and 85 °C) and cooling (after exiting the die) cycle causes a decrease in viscosity during heating, followed by viscosity recovery as the dough cools, stabilizing the printed product’s shape. However, further studies are needed to fully understand the changes in viscosity and structural integrity after extrusion, particularly for formulations with XG and GG [50].

Table 2 provides rheological parameters based on the power law equation (Equation (1)) for various samples measured at different temperatures (25, 55, and 85 °C). The parameters listed are the consistency coefficient (K), the flow behavior index (n), and the coefficient of determination ($R^{2}$). The $R^{2}$ values are close to 0.99 for all samples, indicating the excellent fit of the rheological model to the experimental data.

The K represents the apparent viscosity of the fluid at a unit shear rate. Overall, most samples showed a decrease in the consistency coefficient with an increase in temperature. For the dough formulated with only water (W), the K is primarily influenced by temperature and water concentration. The K values for W samples exhibit the following trends: at 25 °C, K values range from 7886.8 to 20,239.4 Pa·s^n^; and at 85 °C, K values range from 3497.7 to 4693.5 Pa·s^n^. There is a noticeable decrease in K with an increase in temperature, indicating that higher temperatures lead to a lower consistency coefficient. Mirsaeedghazi et al. [51] state that starch gelatinization, gluten cross-linking, or both are considered possible explanations for the thermally induced rheological changes in wheat flour with the addition of only water. Similarly, since our main BW flour also contains starch in its composition, we can attribute the thermo-induced rheological behavior of BW flour to these general mechanisms as well.

For the dough formulated with W + GG, K shows some fluctuation but remains within a narrower range compared to the W + XG samples, and it has higher values than in the W samples. At 25 °C, K values range from 12,579.0 to 16,782.6 Pa·s^n^; at 55 °C, K values range from 4326.6 to 13,255.4 Pa·s^n^; and at 85 °C, K values range from 3152.8 to 5451.4 Pa·s^n^. The K values for the W + GG samples generally decrease with an increase in temperature. The presence of GG stabilizes the consistency to some extent, but the values still fluctuate, indicating that while GG adds some stability, temperature still plays a significant role. The more controlled decrease in K suggests GG may mitigate some of the temperature sensitivity seen in samples with XG. For the dough formulated with W + XG, K exhibits significant variability. At 25 °C, K values range from 12,579.0 to 30,803.2 Pa·s^n^; at 55 °C, K values range from 20,298.3 to 24,346.0 Pa·s^n^; and at 85 °C, K values range from 2890.7 to 18,074.4 Pa·s^n^. The K values for the W + XG samples show a significant decrease with an increase in temperature, which is more pronounced compared to the W or W + GG samples. The highest initial K values suggest a stronger initial consistency, which diminishes sharply as the temperature increases. XG’s high sensitivity to temperature is evident, indicating that precise temperature control is critical when using XG in formulations. XG is more sensitive to temperature changes compared to GG [52].

While all samples show a decrease in consistency with increasing temperature, the extent and variability of this decrease are influenced by the presence and type of additives (XG, GG). Future experiments should aim for more controlled mixing and uniform temperature distribution in the samples to achieve more consistent and reliable results. In this work, all experiments were conducted immediately after mixing GG and XG with water at 1500 RPM, followed by immediate mixing with flour, with a rest time of only 3–5 min before loading the sample into the barrel and a rest time of only 5 min in the barrel. The results shown by Tiwari et al. [53] indicate that the consistency coefficient (K) for GG demonstrates a stable performance regardless of the increases in intensity, while the decrease in K for XG and PG (pectin gum) remains strongly dependent on the intensity level. Therefore, it is necessary to determine the appropriate mixing intensity and stabilization time for XG with water, whereas GG’s consistency is not significantly affected by mixing intensity.

In addition, n indicates the degree of shear-thinning behavior. All samples exhibit n values less than 1, indicating shear-thinning behavior. At 25°, the lowest n values are seen in the XG samples, suggesting strong shear-thinning effects. At 55 °C, the n values for the GG and XG samples show a slight increase, indicating a decrease in shear-thinning behavior as the temperature rises. At 85 °C, the shear-thinning behavior persists but with higher n values than at lower temperatures, and the XG samples still exhibit significant shear-thinning, though less pronounced than at lower temperatures. Generally, the flow behavior index n shows that all samples exhibit shear-thinning behavior, which becomes less pronounced at higher temperatures. This is due to the reduction in intermolecular forces at higher temperatures, making the fluid less viscous. As the temperature rises, the suspension becomes less viscous, but it increases in viscosity when pressurized [54,55].

### 3.3. Effect of Guar Gum and Xanthan Gum on Buckwheat Dough Rheology

**Figure 5:**
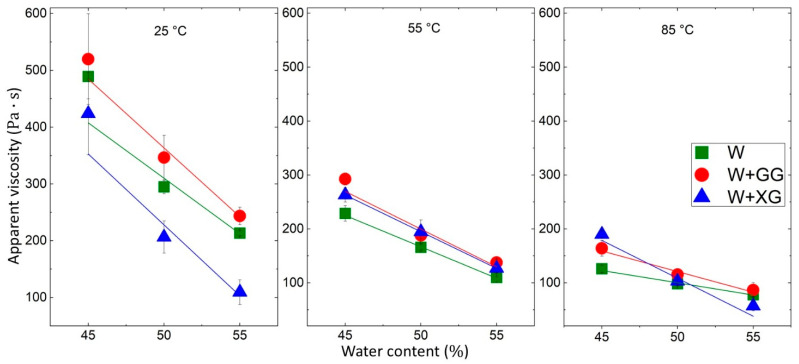
Peak apparent viscosity at a shear rate of 200 s^−1^ versus water content at various temperatures.

Figure 5 illustrates the peak apparent viscosity at a shear rate of 200 s^−1^ versus the water content for different BW dough formulations, measured at the following three temperatures: 25, 55, and 85 °C. Both GG and XG are polysaccharides that, when hydrated, increase dough viscosity by forming a gel-like network [56]. This enhances water retention, thickens the dough, and stabilizes its structure, which are essential for 3D printing.

At 25 °C, GG significantly increases the viscosity of the dough compared to the no gum (W) and XG samples. GG’s strong thickening properties are evident at this lower temperature; at 55 °C, all formulations show a decrease in viscosity compared to 25 °C. However, GG remains the most effective in maintaining higher viscosity at this moderate temperature. At 85 °C, the viscosity continues to decrease for all formulations, and the differences between them are less distinct. Although XG has a much higher molecular weight (1 × 10^6^ to 50 × 10^6^ Da) compared to GG (220,000 to 300,000 Da), the concentration of GG and its interaction with water lead to a more pronounced increase in viscosity. GG’s structure allows for better water trapping and network formation, enhancing its thickening effect [57]. XG’s complex structure and dependence on stirrer speed may not interact as effectively with water under high shear conditions, leading to lower viscosity increases compared to GG at all temperatures and with base dough at room temperature (25 °C) [58,59]. The effect of these additives decreases at higher temperatures, demonstrating the complex interactions between temperature, water content, and additive type on the rheological properties of BW dough.

### 3.4. Shear Rate Distribution in CFD

**Figure 6:**
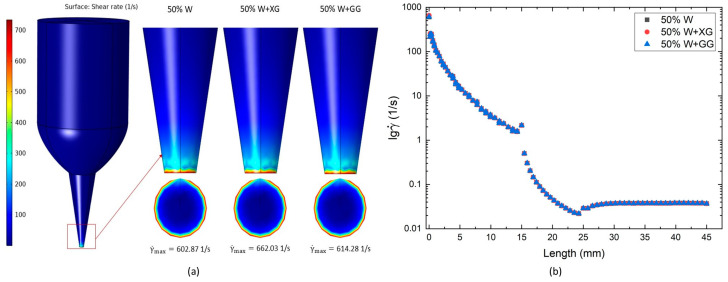
(**a**) Simulated distribution of shear rates; (**b**) shear rate distribution along the centerline of a tube for various dough formulations.

Figure 6a demonstrates that the simulated shear rates of all dough formulations followed a similar pattern. Shear rates were relatively low within the syringe but increased progressively through the nozzle, reaching their peak near the nozzle tip. Comparable results have been reported in other studies [60]. This behavior is attributed to the decreasing diameter of the tube from the syringe to the nozzle tip, which results in an increased fluid velocity and, consequently, higher shear rates, representing the velocity gradients of the dough formulations. The highest shear rates observed were 602.87, 662.03, and 614.28 s^−1^ for 50% W, 50% W + XG, and 50% W + GG, respectively.

The semi-logarithmic plot in Figure 6b illustrates the variation in shear rate along the centerline of a tube for three dough formulations. The y-axis, presented on a logarithmic scale, represents the shear rate, while the x-axis corresponds to the length along the tube’s centerline. The high shear rate at the nozzle outlet caused a reduction in the dough viscosity, suggesting that the dough becomes easier to extrude during the 3D printing process. Then, the plot demonstrates a sharp decrease in shear rate with an increase in length, particularly in the range from 0 mm to approximately 15 mm, indicating an almost exponential decay in all three datasets during the initial phase. In the region between 15 mm and 25 mm, a transition zone is observed, marked by a reduced slope that reflects a slower rate of change in the shear rate. Beyond 25 mm, the shear rate stabilizes and approaches a nearly constant value, regardless of the material composition, as the length further increases up to 45 mm. This distribution suggests that the shear rate is influenced by the geometry of the pipe, which may be gradually tapered. Although minor variations exist between the datasets, the overall trends remain consistent across the different compositions. These findings indicate that despite the addition of different additives, such as XG and GG, the shear rate along the centerline of the pipe follows a similar pattern for all three of the compositions studied.

### 3.5. Viscosity Distribution in CFD

**Figure 7:**
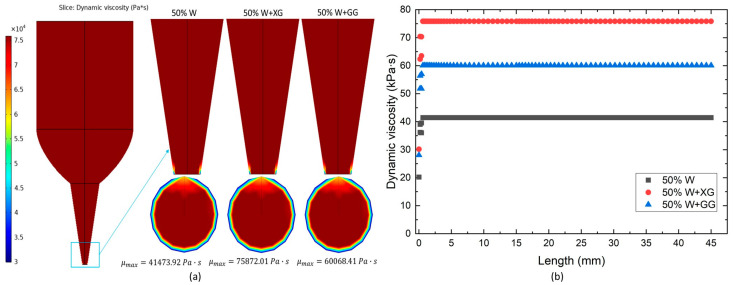
(**a**) Simulated distribution of viscosity; (**b**) apparent viscosity measurements along the central axis for various dough formulations.

As demonstrated in the previous section, shear rates were not uniformly distributed across the entire computational domain, so it is reasonable to expect that the viscosities of the dough formulations were also not uniformly distributed. From Figure 7a, it is evident that the viscosity distributions for all dough formulations were similar. Viscosity was highest within the syringe and gradually decreased towards the nozzle tip. Within the nozzle itself, viscosity was predicted to decrease progressively from the center to the walls.

Figure 7b presents the viscosity values along the centerline of the tube during the extrusion of different dough formulations. The viscosity for all dough types was observed to be highest at the syringe inlet and remained elevated until it reached the nozzle, where it began to decrease near the nozzle tip. The viscosity of the various dough formulations, in descending order, was as follows: 50% W, 50% W + GG, and 50% W + XG. Notably, the provided volumetric flow rate had little effect on viscosity changes with respect to shear rate. This suggests that the given power of the 3D printer may be insufficient for the continuous extrusion of dough [61].

The CFD models in this study assume a no-slip condition at the wall, which is valid for the experimental setup. The 1 mm nozzle diameter, while not narrow, influences rheological behavior by inducing shear-induced particle migration toward the center, creating a viscosity gradient from the wall to the core, as seen in the outlet cross-sectional area (Figure 7a). In contrast, in the study by Fatimi et al. [62] larger diameters (e.g., 1.36 mm and 1.54 mm) show better agreement between theoretical and experimental pressures, as particle depletion effects are minimal. For the 1 mm diameter, CFD models must account for these effects on flow resistance and pressure drop by incorporating accurate viscosity profiles.

### 3.6. Pressure Distribution in CFD

**Figure 8:**
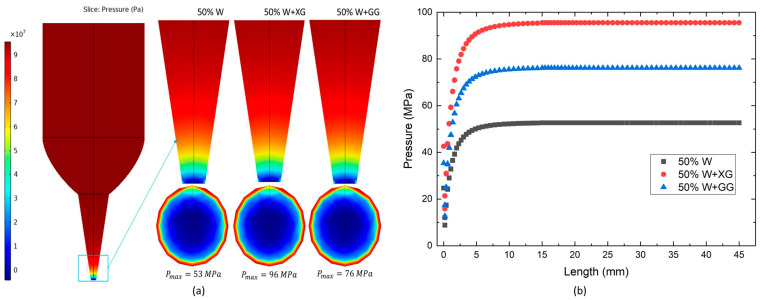
(**a**) Simulated distribution of pressure; (**b**) pressure distribution along the centerline of a tube for various dough formulations.

In 3D food printing, a key factor for ensuring the continuous and smooth extrusion of dough is whether the dough within the syringe can endure the required pressure. As depicted in Figure 8a, the pressure distributions across different dough formulations exhibited a similar pattern. The maximum pressure values, in descending order, were as follows for the grain gels: 50% W + XG (96 MPa), 50% W + GG (76 MPa), and 50% W (53 MPa). If these pressures exceed the maximum force that the printer piston can apply, the dough formulations do not extrude smoothly.

Figure 8b illustrates the simulated pressure values along the centerline of the syringe, from inlet to outlet. These values represent the pressure exerted on the piston [37], and the order of the pressures corresponding to the difficulty of extrusion is as follows: 50% W + XG > 50% W + GG > 50% W. A higher pressure indicates a greater challenge in extruding the dough.

## 4. Conclusions

This study highlights the significant impact of temperature, water content, and the addition of gums (GG and XG) on the rheological properties of BW dough, providing valuable insights for optimizing dough formulations for 3D food printing. The experimental results indicate that increasing water content consistently decreases the apparent viscosity of buckwheat dough, with the 45% *w*/*w* water content exhibiting the highest viscosity. The study also demonstrates that temperature has a noticeable impact on the rheological properties, with higher temperatures generally reducing the dough’s viscosity. The presence of GG stabilizes the consistency of the dough, mitigating some of the temperature sensitivity observed in XG formulations. The consistency coefficient (K) and flow behavior index (n) were highly dependent on temperature, water content, and additive type, with GG formulations showing more controlled decreases in K compared to XG.

The CFD simulation of the extrusion process at 25 °C for the dough formulations (50% W, 50% W + XG, and 50% W + GG) provided valuable insights regarding the shear rate, viscosity, and pressure during syringe-based 3D printing. The shear rate exhibited an initial exponential decrease along the centerline of the tube, stabilizing after 25 mm, with similar trends across all compositions, suggesting geometry is the primary factor influencing shear rate. Viscosity followed a similar pattern for all formulations, being highest near the syringe inlet and decreasing toward the nozzle tip. The addition of XG and GG altered the viscosity profiles slightly. Pressure distribution showed that additives, especially XG, increased extrusion resistance. Overall, the results highlight the critical influence of syringe and nozzle geometry on flow behavior and emphasize the importance of formulation optimization for efficient 3D food printing.

These findings underscore the potential of buckwheat-based formulations in creating customized, nutritious, and sustainable 3D-printed food products. Future research should focus on refining these formulations to enhance printability and stability, contributing to innovations in the food industry and addressing dietary needs and food security challenges. 

## Figures and Tables

**Table 1:** Key attributes of dough formulations—sample designation, water ratios, and gum types—for various formulations.

No	Sample Designation	Water Ratio (% *w*/*w*)	Gum Inclusion
1	45% W	45	No gum
2	50% W	50	No gum
3	55% W	55	No gum
4	45% W + XG	45	XG
5	50% W + XG	50	XG
6	55% W + XG	55	XG
7	45% W + GG	45	GG
8	50% W + GG	50	GG
9	55% W + GG	55	GG

Abbreviations: W—water; XG—xanthan gum; GG—guar gum.

**Table 2:** Rheological properties of BW dough formulations at various temperatures.

*T*, (°C)	Sample Name	$K,\;(Pa\cdot s^{n})$	n	$R^{2}$
25	45% W	20,239.4	0.298	0.99
	50% W	9263.5	0.349	0.99
	55% W	7886.8	0.318	0.99
	45% W + GG	16,782.6	0.344	0.99
	50% W + GG	12,579.0	0.321	0.99
	55% W + GG	14,569.9	0.228	0.99
	45% W + XG	30,803.2	0.191	0.99
	50% W + XG	12,678.9	0.223	0.99
	55% W + XG	19,982.2	0.022	0.98
55	45% W	9416.7	0.298	0.99
	50% W	9599.4	0.234	0.99
	55% W	3827.7	0.329	0.99
	45% W + GG	13,255.4	0.280	0.99
	50% W + GG	5569.8	0.360	0.99
	55% W + GG	4326.6	0.349	0.99
	45% W + XG	20,393.5	0.179	0.99
	50% W + XG	20,298.3	0.123	0.99
	55% W + XG	24,346.0	0.001	0.99
85	45% W	3497.7	0.372	0.99
	50% W	4693.5	0.270	0.99
	55% W	4018.9	0.256	0.99
	45% W + GG	5451.4	0.338	0.99
	50% W + GG	4026.4	0.329	0.99
	55% W + GG	3152.8	0.323	0.99
	45% W + XG	18,074.4	0.139	0.99
	50% W + XG	5730.1	0.239	0.99
	55% W + XG	2890.7	0.259	0.99

Abbreviations: W—water; XG—xanthan gum; GG—guar gum.

## Data Availability

The data presented in this study are available on request from the corresponding author. The data are not publicly available due to privacy restrictions.

## Abbreviations

The following abbreviations are used in this manuscript:

AM	Additive Manufacturing
BW	Buckwheat
CAD	Computer-Aided Design
CFD	Computational Fluid Dynamics
FEM	Finite Element Method
GG	Guar Gum
RPM	Revolutions per minute
XG	Xanthan Gum
